# Molecular Modification of Metadherin/MTDH Impacts the Sensitivity of Breast Cancer to Doxorubicin

**DOI:** 10.1371/journal.pone.0127599

**Published:** 2015-05-19

**Authors:** Zhenchuan Song, Yong Wang, Chao Li, Donghong Zhang, Xinle Wang

**Affiliations:** Department of Breast Center, Fourth Hospital of Hebei Medical University No. 169 Tian Shan Street, Shijiazhuang, 050035, China; Weizmann Institute of Science, ISRAEL

## Abstract

**Background:**

Breast cancer is a leading cause of death in women and with an increasing worldwide incidence. Doxorubicin, as a first-line anthracycline-based drug is conventional used on breast cancer clinical chemotherapy. However, the drug resistances limited the curative effect of the doxorubicin therapy in breast cancer patients, but the molecular mechanism determinants of breast cancer resistance to doxorubicin chemotherapy are not fully understood. In order to explore the association between metadherin (MTDH) and doxorubicin sensitivity, the differential expressions of MTDH in breast cancer cell lines and the sensitivity to doxorubicin of breast cancer cell lines were investigated.

**Methods:**

The mRNA and protein expression of MTDH were determined by real-time PCR and Western blot in breast cancer cells such as MDA-MB-231, MCF-7, MDA-MB-435S, MCF-7/ADR cells. Once MTDH gene was knocked down by siRNA in MCF-7/ADR cells and overexpressed by MTDH plasmid transfection in MDA-MB-231 cells, the cell growth and therapeutic sensitivity of doxorubicin were evaluated using MTT and the Cell cycle assay and apoptosis rate was determined by flow cytometry.

**Results:**

MCF-7/ADR cells revealed highly expressed MTDH and MDA-MB-231 cells had the lowest expression of MTDH. After MTDH gene was knocked down, the cell proliferation was inhibited, and the inhibitory rate of cell growth and apoptosis rate were enhanced, and the cell cycle arrest during the G0/G1 phase in the presence of doxorubicin treatment. On the other hand, the opposite results were observed in MDA-MB-231 cells with overexpressed MTDH gene.

**Conclusion:**

MTDH gene plays a promoting role in the proliferation of breast cancer cells and its high expression may be associated with doxorubicin sensitivity of breast cancer.

## Introduction

Breast cancer is the most common malignancy in women, and mainly affects middle-aged or older women with the age of 40–60 years old. Breast cancer accounts for 23% of malignant tumors in women, and it causes 14% of cancer-related death in women [1]. Currently, the major treatments for breast cancer are comprehensive methods such as surgery, chemotherapy, and radiotherapy. However, because breast cancer is highly metastatic, prone to recurrence, and highly resistant to drugs, the prognosis remains unsatisfactory in spite of comprehensive treatment [2]. Although the initial treatment efficacy may be high, the drug resistance in 90% of breast cancer patients can be developed during disease progression [3, 4]. Both congenital and acquired drug resistance have high impact on the treatments of breast cancer. It is clear that the drug resistance of breast cancer has become a clinical issue that needs urgently to be resolved. Therefore, exploring corresponding strategies that can reverse or reduce the drug resistance of chemotherapy has become a major challenge in oncology.

Metadherin (*MTDH*), also known as *LYRIC*, *AEG-1*, or *3D3*, is expressed in multiple tumors as an oncogene. The protein product of the *MTDH* gene is called metadherin. It is a basic membrane protein containing 582 amino acids, with a molecular weight of 64 kDa, and an isoelectric point of 9.33 [5]. Currently, a large number of experiments have confirmed that the *MTDH* gene can be used as a biomarker to evaluate the prognosis of breast cancer. MTDH might be a pivotal molecule not only in the progression of cancers but also in relation of cancer, innate immunity and inflammation[6]. Previous immunohistochemical analysis of pathological sections from 225 patients with breast cancer has demonstrated high expression of MTDH in 44.4% of cases [7] and the expression level of MTDH is positively correlated with the degree of malignancy [8]. The susceptibility of breast cancer cells to several chemotherapy drugs reveals a significant increase after *MTDH* silencing [9]. When the *MTDH* gene in hepatocellular carcinoma from nude mice is silenced by using lentiviral transfected siRNA, an obviously improved treatment efficacy of 5-fluorouracil and docetaxel is observed [10]. Knockdown of MTDH significantly inhibited proliferation motility and migration of MDA-MB-231[11], and knockdown of endogenous MTDH cells sensitized the MDA-MB-231 cells to TRAIL-induced apoptosis both in vitro and in vivo. Conversely, stable overexpression of MTDH in MCF-7 cells enhanced cell survival with TRAIL treatment[12]. Similarly, the susceptibility of breast cancer cells to multiple chemotherapeutic drugs exhibits a significant enhancement due to the silencing of *MTDH* gene [13].

Currently, many studies on MTDH mainly focus on its expression difference, molecular characterization, signaling pathways, and MTDH impacts in different cancer cells. Although a large number progresses have already been made, the change in the expression of *MTDH* in breast cancer cells with different molecular phenotypes, and its impact on drug resistance to anthracyclines are still unclear. The aim of this study is to determine the change of *MTDH* expression in breast cancer cells with different molecular phenotypes, and its relationship with doxorubicin resistance. Furthermore, in the present study, we would attempt to identify a drug, or an approach that can reverse the clinical resistance of breast cancer cells to doxorubicin through inhibiting *MTDH* gene.

## Materials and Methods

### Cell culture and drug treatments

Breast cancer cells MDA-MB-231and MCF-7 were obtained from Scientific Research Center of the Fourth Hospital of Hebei Medical University, while MDA-MB-435S and MCF-7/ADR were purchasedfromBeijing Silver Amethyst biological medicine technology Co., Ltd. These cells were cultured in RPMI1640 medium with 10% FBS at a 37°C incubator supplemented with 5% CO_2_. The cells were treated with doxorubicin (Zhijiang Haizheng Pharmaceutical Co., LTD, Hangzhou, China) 24 h after seeding into 96-well plates at a density of 1 × 10^5^/well for 100 μL in each well. The cells without doxorubicin treatment were used as the negative control. After 24 h treatment, the cells were harvested and the susceptibility of the cells to doxorubicin was evaluated. Experiments were repeated three times independently.

### Real-time PCR analysis

Total RNA extraction for breast cancer cells was performed by using Trizol (Invitrogen, Shanghai, China) method, and mRNA was reverse-transcribed into stable cDNA. The reaction conditions included 42°C for 60 min, 70°C for 5 min, 4 °C for ∞. Internal control and *MTDH* primers for real-time PCR were obtained from the reference [14] and the forward and reversed primers of MTDH were AAATAGCCAGCCTATCAAGACTC and TTCAGACTTGGTCTGTGAAGGAG. The forward and reversed primers of GAPDH were ACCCACTCCTCCACCTTTG and CTCTTGTGCTCTTGCTGGG. The reaction was conducted under the conditions with 95°C for 30 sec, 95°C for 5 sec, 69°C for 34 sec during 40 cycles. Identity of the PCR-products was controlled by melting curve analysis. Standard curves were prepared from isolated PCR products by serial dilution. Data were normalized towards MTDH expression and statistically analyzed (2^-△△CT^).

### Western blot analysis

Breast cancer cells were collected and lysed to extract total protein. The total protein was quantitatively measured using BCA protein assay kit(GenStar Biosolutions, Beijing, China) according to the manufacturer’s instructions. Equal amounts of protein samples with satisfactory concentrations were loaded into the sample wells of the gel plate. SDS-PAGE (sodium dodecyl sulfate polyacrylamide gel electrophoresis) was performed, while the proteins in samples were separated on the basis of their molecular weights. Then, the target protein in the gel was transferred to the polyvinylidene difluoride (PVDF) membrane through electrophoresis (90 V, 3.5 h). After the electrophoresis, the membrane blot was incubated with the blocking buffer prepared by skimmed milk powder (5% w/v) for 1 h. Then, the membrane blot was sequentially subjected to incubation with primary rabbit anti-human monoclonal MTDH antibodies (ProteintechGroup, Wu Han, China) at 4°C overnight, PBS-T buffer washing for 3 times (20 min for each time), incubation with secondary anti-rabbit antibody (Kang Beiyuan technology, Beijing, China) labeled with horseradish peroxidase at room temperature for 1 h, and the washing with PBS-T buffer for 3 times (20 min for each time) to remove the unbound secondary antibody. Finally, the target protein was visualized with an enhanced chemiluminescence system and normalized towards the GAPDH signal from the same blot.

### The silencing and identification of MTDH gene in MCF-7/ADR cells

The purification and amplification of siRNA sequences was carried out by Wuhan Genesil Biotechnology Co., Ltd (Wuhan, China).

MTDH-siRNA-1: 5’-AATCTCCGGAGCGAGGAACAG-3’

MTDH-siRNA-2: 5’-AACAGAAGAAGAAGAACCGGA-3’

MTDH-siRNA-3: 5’-AACCGCATCATTTCCTGTTGG-3’

HK-siRNA: 5’-GAGUGGGUCUGGGUCUUCCCGUAGA-3’

Cells were seeded into a 6-well plate at a density of 1.5 × 10^5^ cells/well and cultured for 24 h before siRNA transfection. Blank control, negative control, MTDH1, MTDH2, and MTDH3 groups were set up. Lipofectamine 2000 (Invitrogen, Shanghai, China) was used for cell transfection according to the manufacturer’s instructions. RPMI-1640 culture medium containing 100 mL/L serum was used after transfection for 6 h. Total mRNA and protein were extracted after culturing for another 42 h. At 48 h after transfection, *MTDH* mRNA and protein contents were determined by the method of real-time PCR and Western blot in the blank control, negative control, MTDH1, MTDH2, and MTDH3 groups. Experiments were repeated three times independently.

### Transfection of MTDH plasmids into MDA-MB-231 cells

An *MTDH* high expression plasmid pCMV6-Entry with a product number of RC207238 was purchased from Beijing OriGene Technology Co., Ltd.(Beijing, China). The transfection procedures of the plasmid was taken using Lipofectamine 2000 (Invitrogen, Shanghai, China) and the mRNA and protein of MTDH were determinated before and after transfection.

### MTT assay

Breast cancer cells were harvested in the exponential growth phase, and the cell suspension concentration was adjusted so that the cell density was 1 × 10^5^/well for 100 μl in each well. The culture medium was discarded when the cell covered the bottom of each well. Totally 100 μl of doxorubicin(drugs was dissolved by 5 ml 0. 9% sodium chloride injection, and was diluted into five concentrations 0.125 mg/L, 0.25 mg/L, 0.5 mg/L, 1.0 mg/L and 2.0 mg/L by culture medium without serum) was added into each well with 6 replicates. The negative control was administrated with culture media without chemotherapeutic drugs. We found that most breast cancer cells died when the cell was cultured at 48 h. So 10 μl of MTT (Sigma, Shanghai, China) solution was added into each well when the cell was cultured at 24 h, and 100 μl of dimethyl sulfoxide was then added to each well to dissolve the crystal substances completely with low-speed shaking of the cell culture plate for 10 min on a shaker. The absorbance of each well was measured through an optical density (OD) at 490 nm with an enzyme-linked immunometric meter. The IC50 of the breast cancer cell lines was calculated by Improved karber method, and the minmum value of IC50 was 0.93±0.05mg/l. The concentration of 1mg/l was selected to explore the sensitivity of breast cancer to doxorubicin and the inhibitory rate of cell growth was calculated (Cell growth inhibition rate = (1 − experimental group optical density value/control group optical density value) × 100). Experiments were repeated three times independently.

### Examination of apoptosis and cell cycle detection

MDA-MB-231 or MCF-7/ADR cells in the exponential growth phase were harvested and digested by using 0.25% trypsin. Then, the cells were counted and prepared into cell suspension with a cell density of 2.5 × 10^5^ cells/ml in RPMI-1640 media containing 10% fetal bovine serum (FBS). The suspension was well mixed and transferred to a 6-well plate with 2 ml in each well. The cells were then cultured in an incubator containing 5% CO_2_ at 37°C for 18–24 h. When the culture reached the population of 80–90% with even cell growth, the cells were grouped according to the experimental protocols with the consideration of different treatment factors. Then totally 1 ml of propidium iodide (PI) (50 mg/L, Triton-X 100 1.0%) was added into 0.1 ml of cell suspension with the cell density of 1 × 10^5^ cells/ml for staining at 4°C refrigerator for 30 min. The single cell suspension was prepared after filtered with 500 mesh copper sieve. The flow cytometry instrument (BD-Biosciences, Heidelberg, Germany) was adjusted with standard fluorescent microsphere to ensure the CV (coefficient of variation) less than 2%.

MDA-MB-231 or MCF-7/ADR cells (1 × 10^6^ cells) in a six well plates were incubated (37°C, 5% CO_2_) and the cells were grouped according to the experimental protocols with the consideration of different treatment factors. Following trypsinization, cells were washed and centrifuged at 2000 ×g for 10 min and the pellet was resuspended in 1 mL PBS. Fixation was completed by adding 70% cold ethanol at 4°C overnight. The fixed cells were washed with PBS and resuspended in 0.5 mL PBS with propidium iodide (0.05 mg/mL), DNAase free RNAse A (100 μg/mL) and 0.2% Triton X-100. Cells were incubated at 4°C in dark for 30 mins. The cells were analyzed for cell cycle using flow cytometry (BD-Biosciences, Heidelberg, Germany) with an excitation wave length of 488 nm and emission at 670 nm. DNA content was determined by ModFit software (Verity Software House, Topsham, ME), which provided histograms to evaluate cell cycle distribution. Cells treated with the vehicle (DMSO) alone were used as a control.

### Statistical analysis

Statistical analyses were performed with SPSS for Windows 13.0. All continuous variable values were expressed as Mean ± SD (n = 3). Comparison of means between two groups was performed with student t tests. Comparisons of means among multiple groups were performed with one-way ANOVA followed by LSD test (homogeneity of variance) and Games—Howell test (heterogeneity of variance). *P* (probability) < 0.05 was accepted as statistically significant.

## Results

### The expression of MTDH in four different types of breast cancer cell lines and their sensitivity to doxorubicin

**Fig 1:**
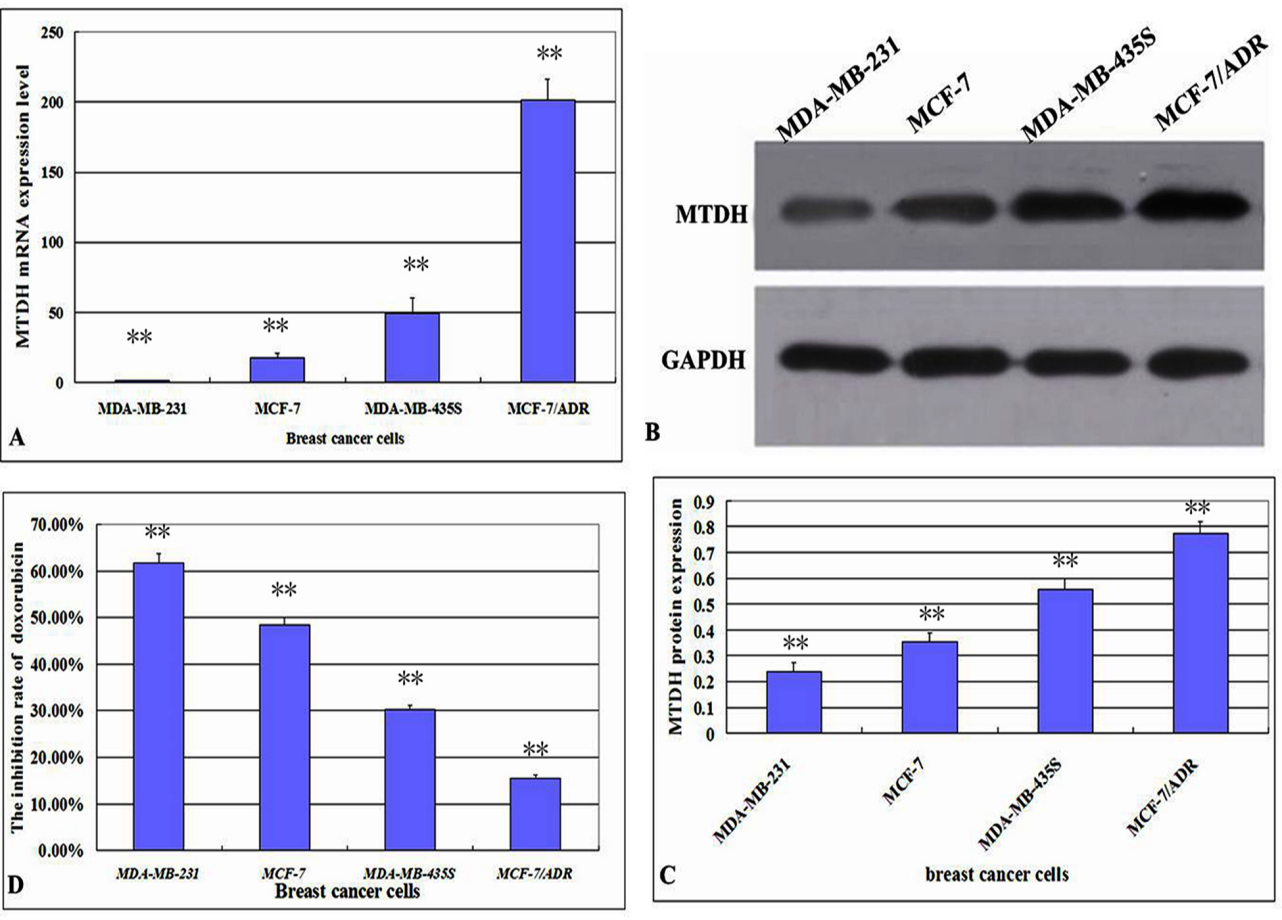
MTDH gene expression in MDA-MB-231, MCF-7, MDA-MB-435S and MCF-7/ADR cells and these cells sensitivity to doxorubicin. (A) The mRNA expression of MTDH determined by real time-PCR. (B, C) The MTDH protein expression was determined by Western Blot. (D) The growth inhibitory rate of breast cancer cells in the presence of doxorubicin determined by MTT assay. Values are expressed as means ±SD of at least three independent experiments, **p<0.01as compared with their respective other group.

We examined the expression of MTDH in four different types of breast cancer cell lines and tested their sensitivity to doxorubicin. The mRNA expression of MTDH gene in breast cancer cells revealed an increasing trend from low to high level, as 1.11 ± 0.10 in MDA-MB-231 cells, 17.58 ± 3.11 in MCF-7 cells, 49.66 ± 10.77 in MDA-MB-435S cells, and 201.74 ± 14.57 in MCF-7/ADR cells (Fig 1A). Consistently, the protein expression levels of MTDH were also found from low to high level, respectively in MDA-MB-231 (0.238±0.035), MCF-7 (0.355±0.036), MDA-MB-435S (0.555±0.045), MCF-7/ADR (0.773±0.045) (Fig 1B and 1C). However, when the cells were treated with doxorubicin for 24 h, the cell growth was inhibited in a reverse manner. The most obvious inhibition (61.69%) was observed in MDA-MB-231 cell line, but the least inhibition (15.51%) was observed in MCF7/ADR cell line, indicating that the expression level of MTDH is negatively associated with the susceptibility of breast cancer cells to doxorubicin. Therefore, the higher MTDH-expressed breast cancer cell line displayed the more resistance to doxorubicin (Fig 1D).

### MTDH knockdown promotes the sensitivity of MCF-7/ADR cells to doxorubicin

**Fig 2:**
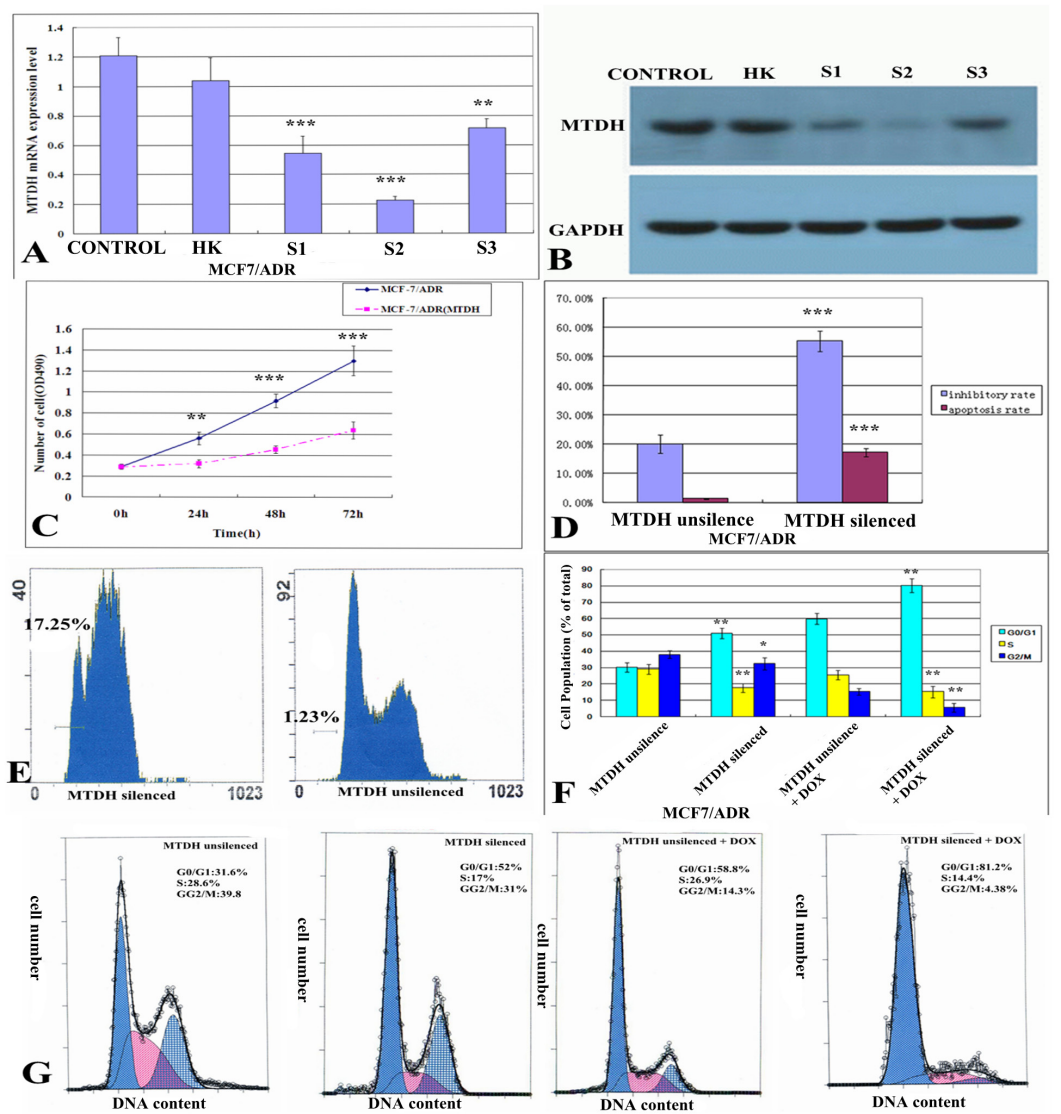
The effect of MTDH gene silencing on the clinical sensitivity of doxorubicin. (A) The mRNA expression of MTDH determined by real time-PCR after siRNA interference for 48 h (MCF-7/ADR cells are divided into 5 groups including blank control group 1.026±0.126, negative control group 1.038±0.158, MTDH1 0.545±0.116, MTDH2 0.227±0.021 and MTDH3 group 0.717±0.060) showed that MTDH2 siRNA was the best interference on MTDH gene silence. (B) Protein expression of MTDH determined by western blot after siRNA interference for 48 h (MCF-7/ADR cells are divided into 5 groups including blank control group 1.419±0.012, negative control group 1.327±0.094, MTDH1 0.204±0.029, MTDH2 0.037±0.01 and MTDH3 0.280±0.005 groups) showed that MTDH2 siRNA was also the best interference on MTDH gene silence. (C) The cell growth curves showed the MCF-7/ADR cell growth was inhibited after the cells were interfered in MTDH2 group. (D) The inhibitory rates of MCF-7/ADR cells measured by a MTT assay subjected to 1 mg/L doxorubicin treatment for 24 h were (20.10 ± 3.23)% and (55.40 ± 3.49)% in MTDH gene unsilence group and silenced group respectively (*P* = 0.001). (D, E) The apoptosis rates of MCF-7/ADR cells measured by FCM subjected to 1 mg/L doxorubicin treatment for 24 h were (1.36 ± 0.14)% and (17.21 ± 1.35)% in MTDH gene unsilence group and silenced group respectively (*P* = 0.000). **(**F, G) The cell cycle measured by FCM and showed that knockdown of MTDH resulted in accumulation in the G0/G1 phase and reduction of S and G2/M phase and after treatment with doxorubicin cell cycle was induced arrest more at G0/G1 phase and reduction of S and G2/M phase in MTDH silenced group compared to the unsilenced group. Values are expressed as means ± SD of at least three independent experiments, *p<0.05, **p<0.01 and ***p<0.001 as compared with their respective unsilence group.

In order to confirm the role of MTDH in the sensitivity of breast cancer cells to doxorubicin, we designed three different short-hairpin RNA constructs (MTDH-siRNA1, MTDH-siRNA2, and MTDH-siRNA3) to knock down MTDH in MCF-7/ADR cell line. The expression levels of both mRNA and protein of MTDH were determined after the transfection of different siRNA constructs for 48 h. Among these constructs, the MTDH-siRNA2 significantly down-regulated both mRNA and protein expression levels of MTDH in MCF-7/ADR cell line (Fig 2A and 2B). Therefore, MTDH-siRNA2 construct was selected to knockdown MTDH. Next, we assessed the cell viability and cell growth of the MTDH knockdown cells by MTT assay. As expected, knock down of MTDH could significantly inhibite the cell growth of MCF-7/ADR cells after the MTDH knocked down for 24, 48 and 72 h compared to the without knockdown group(Fig 2C), and knockdown of MTDH resulted in accumulation in the G0/G1 phase and reduction of S and G2/M phase cell (Fig 2F and 2G). Furthermore, to investigate whether the MTDH knockdown cells become more susceptible to doxorubicin, we assessed the cell growth, cell cycle and apoptosis in MCF-7/ADR cells. After doxorubicin treated for 24h the inhibitory rates of MCF-7/ADR cells were (20.10 ± 3.23)% and (55.40 ± 3.49)% in MTDH gene unsilence group and silenced group respectively (Fig 2D), and the apoptosis rate was (1.36 ± 0.14)% and (17.21 ± 1.35)% in MTDH gene unsilence group and silenced group respectively(Fig 2D and 2E). Furthermore, we also found that treatment with doxorubicin induced cell cycle arrest more at G0/G1 phase and reduction of S and G2/M phase in MTDH silenced group compared to the unsilenced group (Fig 2F and 2G). Indeed, a higher cell growth and apoptotic rate of MTDH knockdown cells was observed when compared with that of the control cells, indicating that the reduced MTDH expression in MCF-7/ADR cells sensitized the cells to doxorubicin.

### Overexpression of MTDH enhances the resistance of MDA-MB-231 cells to doxorubicin

**Fig 3:**
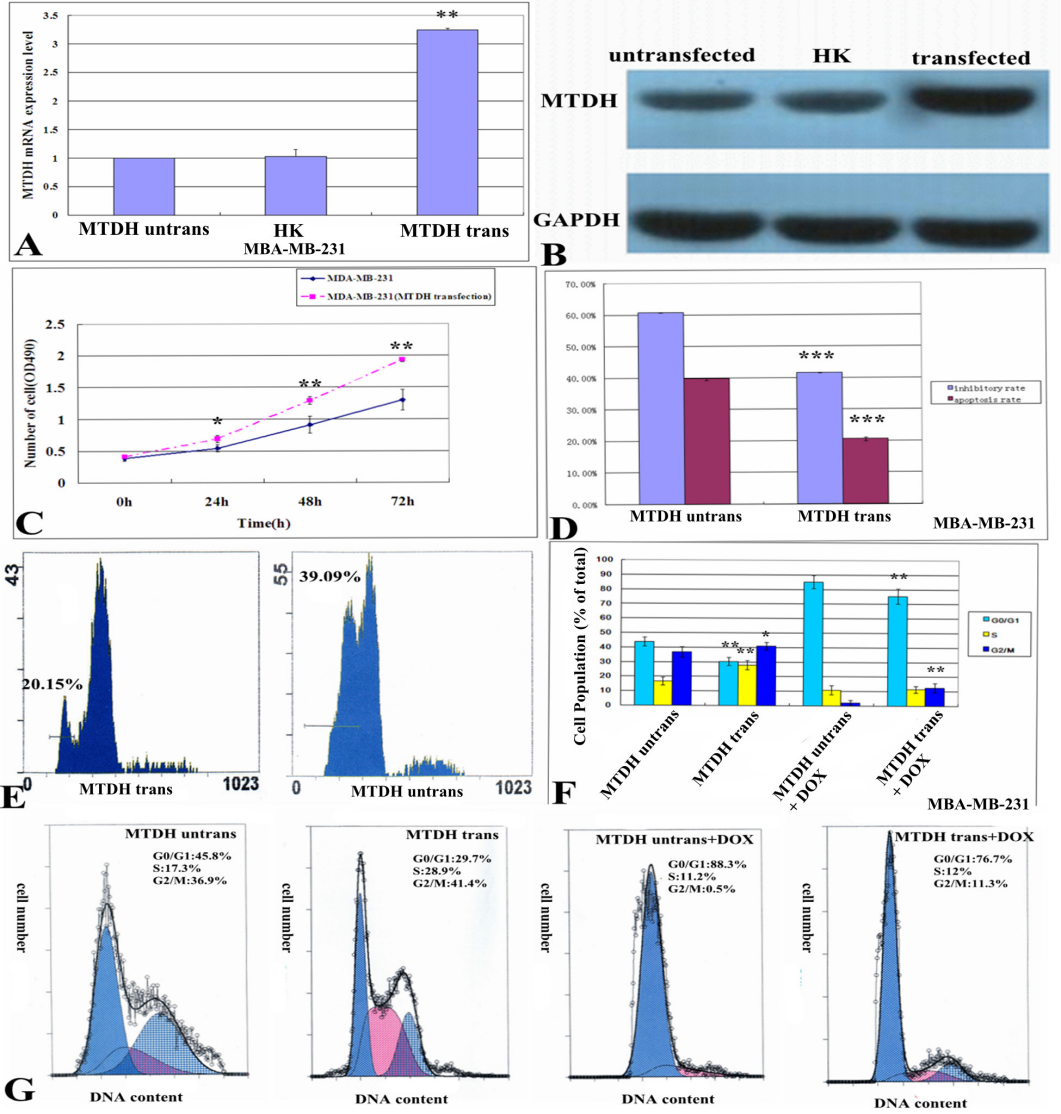
Effect of MTDH gene overexpression on clinical sensitivity of doxorubicin. (A) MTDH mRNA expression determined by real time-PCR after MTDH plasmid was transfected for 48 h in MDA-MB-231 cells (untransfected group 1.00±0.00, blank plasmid group 1.03±0.12 and MTDH transfected group 3.25±0.02). (B) MTDH protein expression determined by western blot after MTDH plasmid was transfected for 48 h in MDA-MB-231 cells (untransfected group 0.48±0.07, blank plasmid group 0.50±0.06 and MTDH transfected group 1.27±0.08). (C) The cell growth curves showed the MDA-MB-231 cell growth was promoted after MTDH gene was transfected into cells. (D) The inhibitory rates measured by a MTT assay of MDA-MB-231 cells subjected to 1 mg/L doxorubicin treatment for 24 h were (60.72 ± 0.01)% and (41.68 ± 0.03)% before and after transfection of MTDH plasmid (*P* = 0.000). (D, E) The apoptosis rates of MDA-MB-231 cells measured by FCM subjected to 1 mg/L doxorubicin treatment for 24 h were (39.76 ± 0.44)% and (20.59 ± 0.59)% before and after transfection of MTDH plasmid (*P* = 0.000). **(**F, G) In the MTDH transfected group, the cell cycle measured by FCM and showed a increase in the proportion of cells in the S and G2/M phase and decrease in cells in the G0/G1 phases compared to the untransfected group. When treated with doxorubicin, the cell cycle arrest more at G0/G1 phase and reduction of G2/M phase in MTDH untransfected group compared to the transfected group. Values are expressed as means ± SD of at least three independent experiments, *p<0.05, **p<0.01 and ***p<0.001 as compared with their respective untransfected group.

In order to further validate the role of MTDH in the resistance of breast cancer cells to doxorubicin, the MDA-MB-231 cell line with overexpressed MTDH revealed the enhanced expression of both mRNA and protein levels (Fig 3A and 3B). In addition, the overexpression of MTDH could significantly promote the cell growth of MDA-MB-231 cells after the MTDH plasmid transfection for 24, 48 and 72 h (Fig 3C), and the cell cycle showed accumulation in the S and G2/M phase and reduction of G0/G1phase (Fig 3F and 3G). After gene transfection and 1 mg/L doxorubicin treated for 24 h, the inhibitory rate of cell growth became lower in MTDH transfected group (41.68%) when compared with pre-transfected group (60.72%)(Fig 3D); meanwhile, the apoptotic rate of the cells also revealed a reduction from 39.76% to 20.59% (Fig 3D and 3E) and the cell cycle arrest more at G0/G1 phase and reduction of G2/M phase in MTDH untransfected group compared to the transfected group (Fig 3F and 3G). Taken together, these results strongly suggest that MTDH expression contributes to the resistance of breast cancer cells to doxorubicin.

## Discussion

As a recognized cancer gene, *MTDH* plays important roles in the onset and development of breast cancer and many other tumors. MTDH can affect the behavior of tumors by regulating multiple signaling pathways and expression levels of related genes. The major signaling pathways included NF-κB, PI3K/AKT/MAPK and Wnt/β-catenin. MTDH also can affect biological behavior of tumors, such as proliferation, angiogenesis, invasion and metastasis, and drug resistance through these signaling pathways. Recently study showed MTDH exon 11 skipping variant can be considered a major promalignant factor for ovarian cancer[15]. Therefore, in order to clarify the relationship between *MTDH* gene and chemotherapeutic susceptibility of breast cancer, we have conducted this study at the cellular level.

Our studies have demonstrated that MTDH expression in 4 types of breast cancer cells was different, and revealed an ascending order as MDA-MB-231 (ER-, PR-, HER-2-), MCF-7 (ER+, PR+, HER-2-), MDA-MB-435S (ER-, PR-,) and MCF-7/ADR. Thus, it can be concluded that MTDH expressions in breast cancer cells are different due to different molecular phenotypes, and the overexpression of MTDH may be associated with the invasiveness and drug resistance of breast cancer cells. Similarly, previous investigation has shown that suppressing *MTDH* can increase the susceptibility of breast cancer cells to chemotherapeutic drugs and stressors [16]. Therefore, the overexpression of *MTDH* is related to drug susceptibility in some cancer cells.

In the present study, *MTDH* expression in breast cancer MCF-7/ADR cells was down-regulated by siRNA interference, and cell proliferation was obviously inhibited after silencing. Our reports also shown that knockdown of MTDH resulted in accumulation in the G0/G1 phase and reduction of S and G2/M phase cell. The inhibitory rate and apoptosis rate of MCF-7/ADR cells from the MTDH group after 24 h treatment by 1 mg/L doxorubicin revealed an obvious decrease and induced cell cycle arrest more at G0/G1 phase and reduction of S and G2/M phase when compared to the control group, which indicating that down-regulating MTDH can inhibit the proliferation of human breast cancer MCF-7/ADR cells, and increase the susceptibility of breast cancer cells to doxorubicin. The molecular mechanism might involve that the down-regulation of MTDH expression significantly increase the activity of PI3K/AKT signaling pathway, cause the elevated expression of FOXO3a, and eventually lead to cell apoptosis[17]. MTDH also affects the multidrug resistance gene (*MDR1*) through this signal pathway, thus causing the resistance to chemotherapeutic drugs [18]. Microarray analysis in breast cancer cells revealed that knockdown of MTDH led to decreased expression of chemoresistance genes ALDH3A1, MET, HSP90, and HMOX1 and increased expression of proapoptotic genes BNIP3 and TRAIL [16].

Up-regulating *MTDH* expression can promote the proliferation of many tumor cell lines including esophageal cancer, gastric cancer, glioma, and breast cancer [16, 19–21]. Many chemotherapeutic drugs can induce apoptosis to certain degrees, and the susceptibility of tumor cell apoptosis might be an important factor that affects the chemotherapeutic efficacy [22].This study further confirmed that up-regulation of MTDH could promote the growth of tumor cells and increase their resistance to doxorubicin.

## Conclusions

In summary, *MTDH* is a proto-oncogene expressed differentially in breast cancer cells with different molecular features. Down-regulation of *MTDH* expression can reduce the proliferation of breast cancer cells and increase their susceptibility to doxorubicin.
